# Lutein/zeaxanthin isomers regulate neurotrophic factors and synaptic plasticity in trained rats

**DOI:** 10.3906/sag-2101-264

**Published:** 2021-08-30

**Authors:** Cemal ORHAN, Füsun ERTEN, Beşir ER, Mehmet TUZCU, Nurhan ŞAHİN, Öznur Ece DURMAZ KURŞUN, Vijaya JUTURU, Kazim ŞAHİN

**Affiliations:** 1 Department of Animal Nutrition, Faculty of Veterinary Medicine, Fırat University, Elazığ Turkey; 2 Department of Veterinary Science, Pertek Sakine Genç Vocational School, Munzur University, Tunceli Turkey; 3 Department of Biology, Faculty of Science, Fırat University, Elazığ Turkey; 4 Department of Pharmacy Services, Vocational School of Kovancılar, Fırat University, Elazığ Turkey; 5 Research and Development, OmniActive Health Technologies Inc., Morristown, NJ USA

**Keywords:** Lutein, zeaxanthin, exercise, oxidative stress, synaptic proteins

## Abstract

**Background/aim:**

This study was conducted to elucidate the effects of lutein/zeaxanthin isomers (L/Zi) on lipid metabolism, oxidative stress, NF-κB/Nrf2 pathways, and synaptic plasticity proteins in trained rats.

**Materials and methods:**

Wistar rats were distributed into four groups: 1) control, 2) L/Zi: rats received L/Zi at the dose of 100 mg/kg by oral gavage, 3) exercise, 4) exercise+L/Zi: rats exercised and received L/Zi (100 mg/kg) by oral gavage. The duration of the study was eight weeks.

**Results:**

Exercise combined with L/Zi reduced lipid peroxidation and improved antioxidant enzyme activities of muscle and cerebral cortex in rats (p < 0.001). In the Exercise + L/Zi group, muscle and cerebral cortex Nrf2 and HO-1 levels increased, while NF-κB levels decreased (p <0.001). Also, L/Zi improved BDNF, synapsin I, SYP, and GAP-43 levels of the cerebral cortex of trained rats (p < 0.001). The highest levels of BDNF, synapsin SYP, and GAP-43 in the cerebral cortex were determined in the Exercise+L/Zi group.

**Conclusion:**

These results suggested that exercise combined with L/Zi supplementation might be effective to reduce neurodegeneration via improving neurotrophic factors and synaptic proteins, and oxidative capacity in the cerebral cortex.

## 1. Introduction

Regular exercise is linked with preventing chronic illnesses such as obesity, diabetes, cardiovascular diseases (CVD), dyslipidemia, and depression [1,2]. It may also stimulate neurogenesis and improve learning and mental performance, new hippocampal cell count, and brain plasticity [3]. Moreover, regular exercise stimulates adaptive responses through the activation of signal transduction pathways, including nuclear factor kappaB (NF-κB) and nuclear factor (erythroid-derived 2)-like 2 (Nrf2) pathways, which rapidly rises in endogenous antioxidant and oxidative DNA damage-repairing systems, thus being useful for the chronic diseases [4]. Regular exercise has been reported to improve neurotrophic factors such as brain-derived neurotrophic factor (BDNF) in the hippocampus, the most important center for learning and memory [5].

Xanthophyll macular pigments lutein and zeaxanthin are isomers that change by the locality of a single, double bond [6]. Humans cannot synthesize lutein and zeaxanthin isomers; for this reason, these nutrients should be taken from natural nutritional sources or supplements [6]. Numerous lutein and zeaxanthin preparations available on the market are extracted from the marigold flower (*Tagetes erecta L.*). Lutein and zeaxanthin are potent antioxidants and, at the same time, act like high-energy blue light filters. It has been reported that these xanthophylls are protective against photo-induced oxidative damage, particularly in exposed tissues such as the skin and eyes [7]. In addition to high concentrations in the eye, L and Z constitute about 66%–77% of the total carotenoid level in the double frontal lobe and visual processing region of the brain [7,8]. Macular carotenoids lutein and zeaxanthin concentrations within the retina seem to be powerfully related to measures of lutein and zeaxanthin in the cerebellum, and the macular pigment optical density has also been linked by criteria such as cognition [9], balance time, and reaction time [10] facilitated by the brain.

Recent studies have shown that lutein/zeaxanthin isomers (L/Zi) regulate genes involved in oxidative stress and inflammation, such as Nrf2 and NF-κB, enhancing retinal injury [11,12]. However, an animal study revealed that supplementation of L/Zi, regular exercise training, or the combination of both was not reported with any improvement in brain and muscle function by regulation of transcription and neurotrophic factors and synaptic proteins. Therefore, we investigated the effects of regular exercise, L/Zi supplementation, or both on lipid metabolism changes, oxidative stress, and muscle and cerebral cortex Nrf2, NF-κB, BDNF, and synaptic proteins in rats. We hypothesized that L/Zi supplementation combined with regular exercise training could modulate the Nrf2, NF-κB, BDNF, and synaptic proteins in the cerebral cortex of rats to a greater extent than either L/Zi supplementation or exercise alone.

## 2. Material and methods

### 2.1. Animals and groups

After two weeks of acclimatization, twenty-eight Wistar rats (8 weeks old, 180–200g) were kept in a laboratory condition (22 °C: 12h light/12h dark) and fed with a standard diet (Table 1). All the study processes were approved by the Ethics Committee of the Animal Experiments of Fırat University (Elazığ, Turkey; 2014/125-235). Rats were divided into four groups: 1) sedentary control: rats received standard diet alone; 2) L/Zi: sedentary with supplementation of L/Zi at the dose of 100 mg/kg BW for eight weeks, 3) exercise: rats received standard diet and exercised regularly for eight weeks, and 4) exercise+L/Zi: exercised with supplementation of L/Zi at the dose of 100 mg/kg BW for eight weeks. Rats in the vehicle-treated control group were administered an equal volume of the vehicle (corn oil). The product L/Zi (Lutemax 2020) from Marigold flowers (T. erecta L) was supplied by OmniActive Health Technologies Ltd. (Mumbai, India). The product was produced by natural saponification and thermal isomerization reaction containing a xanthophyll extract such as marigold oleoresin. The L/Zi sample contains (Batch: 00000062612) 81.34% carotenoids with 66.63% lutein and 14.22% zeaxanthin isomers. The dose (100 mg/kg) used in the study was selected based on the amount used in earlier studies in rodents [13,14]. The experiment lasted eight weeks.

**Table 1:** Composition of the basal diet.

Ingredient	%
Maize	26.00
Wheat	14.00
Vegetable oil	3.00
Soybean meal, 48% CP	33.10
Sunflower meal, 30% CP	8.00
Wheat bran	7.00
Molasses	5.00
Limestone	0.80
Salt	0.80
DL-Methionine	0.80
Dicalcium phosphate	1.20
Vitamin and mineral premix1	0.30
Analysis (%)	
Crude protein	24.27
Ether extract	4.55
Crude cellulose	4.04
Ash	6.91
Ca	0.75
P	0.41

aThe vitamin-mineral premix provides the following (per kilogram): all-trans-retinyl acetate, 1.8 mg; cholecalciferol, 0.025 mg; all-rac-a-tocopherol acetate, 12.5 mg; menadione (menadione sodium bisulfate), 1.1 mg; riboflavin, 4.4 mg; thiamine (thiamine mononitrate), 1.1 mg; vitamin B6, 2.2 mg; niacin, 35 mg; calcium pantothenate, 10 mg; vitamin B12, 0.02 mg; folic acid, 0.55 mg; d-biotin, 0.1 mg; manganese (from manganese oxide), 40 mg; iron (from iron sulfate), 12.5 mg; zinc (from zinc oxide), 25 mg; copper (from copper sulfate), 3.5 mg; iodine (from potassium iodide), 0.3 mg; selenium (from sodium selenite), 0.15 mg; choline chloride, 175 mg.

### 2.2. Training protocol

The animal was exercised at a motorized treadmill with a stimulus grid (May-TME, Commat Inc, Ankara, Turkey), and the training period was given in detail as described previously [4]. 

### 2.3. Sample collection

At the end of the experimental phase of the study, animals were slaughtered by cervical dislocation under anesthesia with intraperitoneal injections of a mixture of xylazine (7 mg/kg) and ketamine (70 mg/kg). Then, blood, gastrocnemius muscles, and whole-brain samples were taken. Serum samples were separated by centrifuge and kept at –80 °C. The tissue samples were kept in a freezer at –80 °C until use.

### 2.4. Laboratory assays

Serum biochemical parameters [glucose, total cholesterol, creatinine and urea, and aminotransferases (AST and ALT)] were detected by a biochemistry analyzer device (Samsung Electronics Co., Suwon, Korea). Tissue lutein and zeaxanthin were detected by HPLC (Shimadzu, Kyoto, Japan) modified from earlier methods [15] and has been defined in detail [16,17]. Tissue malondialdehyde (MDA) levels were also determined by HPLC, consisting of Shimadzu UV- vis SPD-10 AVP detector and C18-ODS-3 column, based on the earlier defined method [18]. SOD, CAT, and GSH-Px activities of muscle and brain tissue were measured by the ELISA kits (Cayman Chemical, Ann Arbor, MI, USA) according to the manufacturer’s guidelines.

### 2.5. Western blot assays

Tissue proteins were evaluated by the Western blot method [19]. The samples were homogenized in phosphate-buffered saline (PBS), containing the protease inhibitor cocktail [19]. The homogenization buffer was added to the homogenates, and the mixture was centrifuged to remove the residues of the cells and separate the total protein. The protein was electrophoresed on a 10% SDS-PAGE and then transferred to nitrocellulose membranes. Membranes were blocked with 1% BSA for 1 h prior to administration of the primary antibody and incubated overnight with primary antibodies against NF-κB, Nrf2, HO-1, BDNF, growth-associated protein-43 (GAP-43), synapsin, and synaptophysin (SYP) (Abcam, Cambridge, UK) were diluted (1:1000) in the same buffer containing 0.05% Tween-20. Western blot normalization was done by β-actin as a control protein. Levels of protein were measured densitometrically in samples using an image analysis system. 

### 2.6. Statistical analysis

Data were noted as mean ± SE. The sample size is based on a power of 85% to achieve a p value of 0.05. Normality of the data was tested with Shapiro–Wilk test. All the parameters showed normal distribution. A parametric analysis of variance (ANOVA) was done, and Tukey’s multiple comparisons were used as a post hoc test to notice changes among the groups. The analyses were done with the program SPSS (IBM SPSS, Version 22.0; Chicago, IL, USA). Statistical significance for the data was defined as significant for probability values less than p < 0.05.

## 3. Results

### 3.1. Biochemical evaluation

There was no difference in serum glucose concentration between all groups (Table 2, p > 0.05). Exercise training significantly reduced serum triglycerides and lactate levels in rats in comparison to the control group (p < 0.0001). The combination of L/Zi and exercise exerted potent inhibitory effects on serum total cholesterol, triglycerides p < 0.05), and lactate levels (p < 0.001) in comparison to exercise-only group and control group (p < 0.001). In exercise+L/Zi rats, serum lactate concentration decreased by 33.6% and 14.5% in comparison with normal and exercised rats, respectively (p < 0.0001). Muscle and brain lutein and zeaxanthin concentrations were higher in the L/Zi treatment rats than in control rats (p < 0.0001). However, there were no alterations for the tissue lutein and zeaxanthin levels between the control and exercise groups (p > 0.05).

**Table 2:** The effects of the Lutein/Zeaxanthin isomers (L/Zi) with regular exercise on serum glucose, total cholesterol (TC), triglycerides (TG) and lactate levels, and tissue lutein and zeaxanthin in rats.

Items	Groups				--p--
	Control	L/Zi	Exercise	Exercise+L/Zi	
Glucose, mmol/L	5.64 ± 0.35	5.63 ± 0.27	5.07 ± 0.23	5.13 ± 0.34	0.400
TC, mmol/L	1.94 ± 0.02a	1.93 ± 0.03a	1.91 ± 0.02a	1.75 ± 0.03b	0.0001
TG, mmol/L	1.17 ± 0.08a	1.16 ± 0.05a	0.95 ± 0.02b	0.85 ± 0.01b	0.0001
Lactate, mmol/L	1.07 ± 0.03a	1.03 ± 0.04a	0.83 ± 0.02b	0.71 ± 0.03b	0.0001
Muscle, mg/g					
Lutein	0.226 ± 0.00b	0.301 ± 0.011a	0.217 ± 0.008b	0.298 ± 0.007a	0.0001
Zeaxanthin	0.115 ± 0.01b	0.168 ± 0.014a	0.112 ± 0.007b	0.173 ± 0.010a	0.008
Brain, mg/g					
Lutein	0.107 ± 0.01b	0.169 ± 0.009a	0.110 ± 0.005b	0.166 ± 0.008a	0.003
Zeaxanthin	0.031 ± 0.00b	0.092 ± 0.004a	0.034 ± 0.003b	0.088 ± 0.004a	0.0001

Data are presented as means and standard error. Different superscripts (a–c) indicate group mean differences (p < 0.05).

### 3.2. Muscle and cerebral cortex oxidative stress and antioxidant enzymes

**Figure 1:**
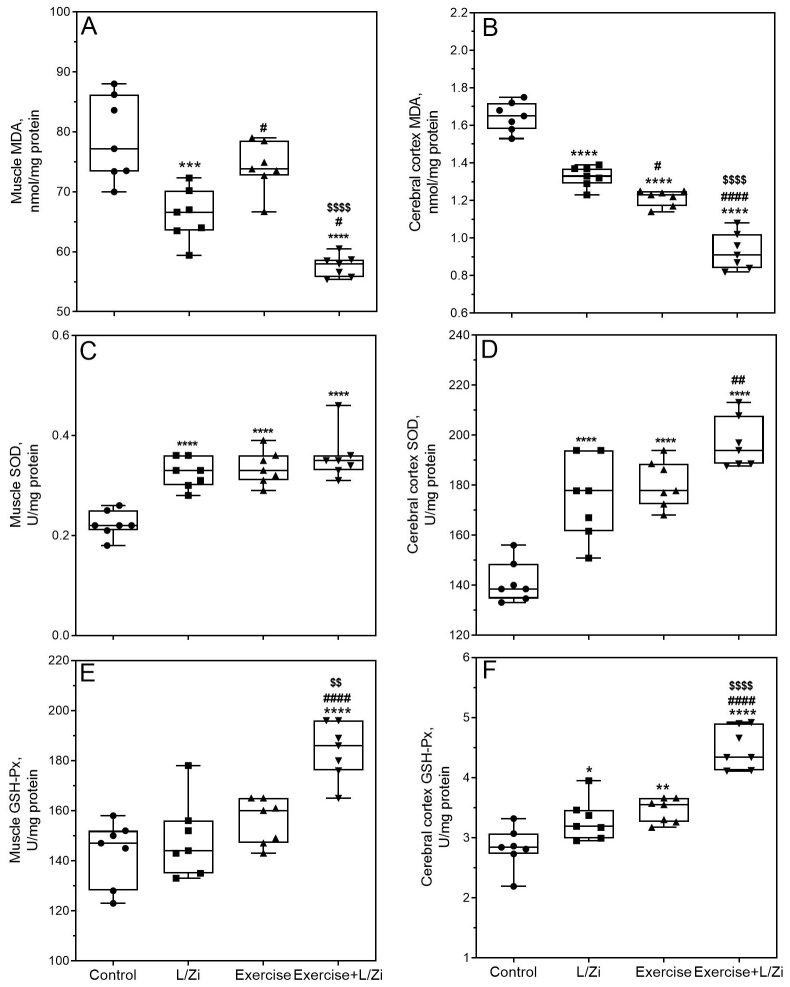
The effects of the lutein/zeaxanthin isomers (L/Zi) with regular exercise on the muscle and cerebral cortex level of malondialdehyde (MDA), superoxide dismutase (SOD), and glutathione peroxidase (GSH-Px) in rats. The level of muscle MDA (Panel A), muscle SOD (Panel C), and muscle GSH-Px (Panel E), cerebral cortex MDA (Panel B), cerebral cortex SOD (Panel D), and cerebral cortex GSH-Px (Panel F) shown in various groups. Each plot represents the median and values (Min to Max. Shown all points). ANOVA and Tukey’s post-hoc tests were used for comparing the results among different groups. Statistical significance between groups is shown by * p < 0.05; ** p < 0.01; *** p < 0.001; **** p < 0.0001 compared as control group, # p < 0.05; ## p < 0.01; ### p < 0.001; #### p < 0.0001 compared as L/Zi group, $ p < 0.05; $$ p < 0.01; $$$ p < 0.001; $$$$ p < 0.0001 compared as exercise group.

Levels of MDA in muscle and cerebral cortex decreased by 16.1% (p < 0.001) and 19.4% (p < 0.0001) in L/Zi rats compared to sedentary rats (Figures 1A, 1B). In exercise + L/Zi rats, muscle MDA levels reduced by 26.9% compared to control rats and 22.3% compared to exercised rats (p < 0.0001). Cerebral cortex MDA levels showed a similar change (43.6% and 23.1%; p < 0.0001). In exercised animals, muscle and cerebral cortex SOD activities increased by 54.5% and 27.8% compared to the control animals (Figure 1C, D; p < 0.0001). 

Similarly, muscle and cerebral cortex GSH-Px activities increased by 8.7% and 21.9% in exercised rats compared to the control animals (Figure 1E, F; p < 0.0001 and p < 0.01). The reduction in MDA levels and increases in SOD and GHS-Px activities of muscle and cerebral cortex in response to exercise+L/Zi treatment were more remarkable than the other treatments (p < 0.001; Figure 1).

### 3.3. Muscle and cerebral cortex protein levels

**Figure 2:**
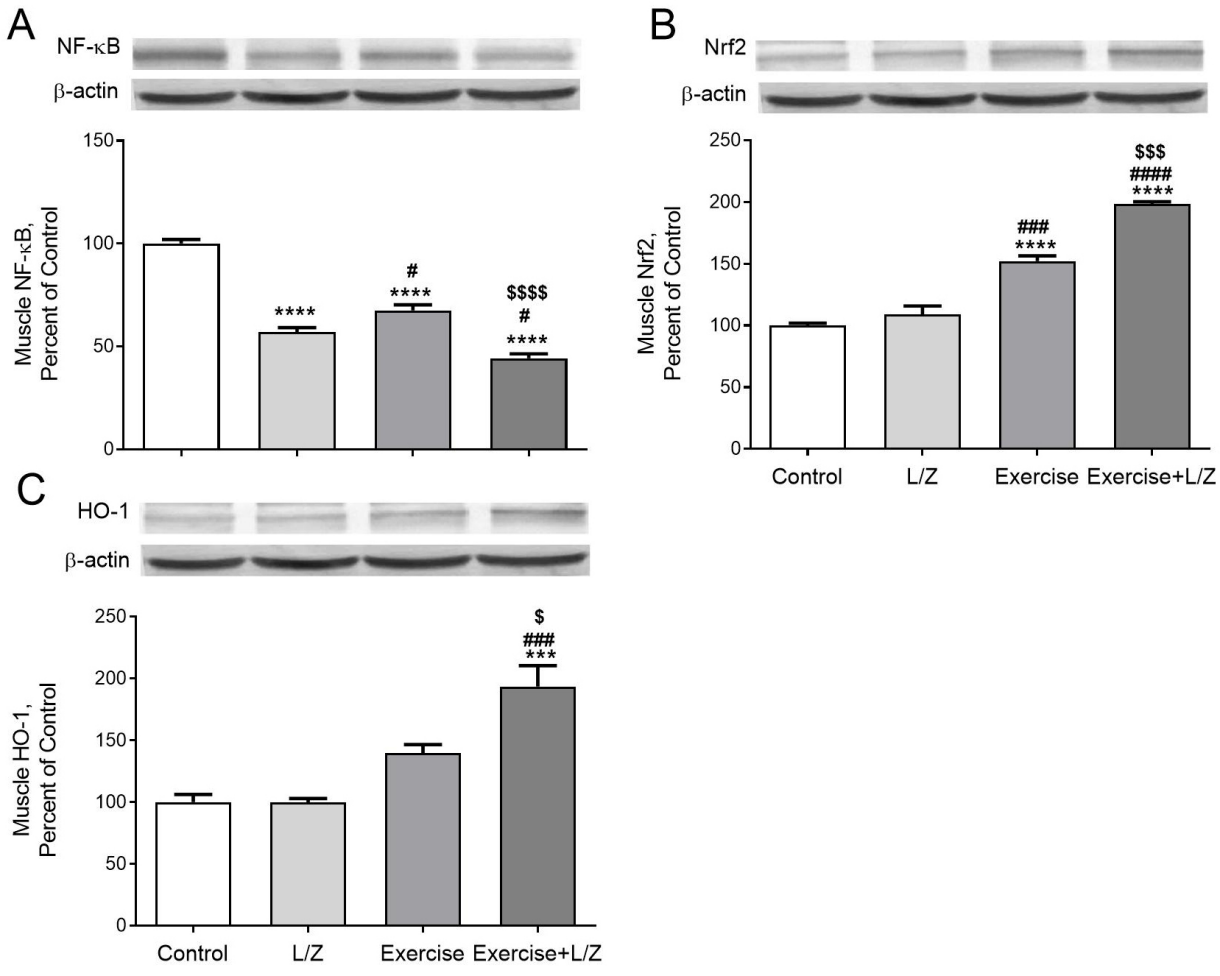
The effects of the lutein/zeaxanthin isomers (L/Zi) with regular exercise on muscle level of NF-kB, Nrf2, and HO-1 in rats. The level of NF-kB (Panel A), Nrf2 (Panel B), and HO-1(Panel C) is shown in various groups. Data are expressed as a ratio of the normal control value (set to 100%). Each bar represents the mean and standard error of the mean. The intensity of the bands shown in the band was quantified by densitometric analysis, and β-Actin was included to ensure equal protein loading. Blots were repeated at least 3 times (n = 3). ANOVA and Tukey’s post-hoc tests were used for comparing the results among different groups. Statistical significance between groups is shown by * p < 0.05; ** p < 0.01; *** p < 0.001; **** p < 0.0001 compared as control group, # p < 0.05; ## p < 0.01; ### p < 0.001; #### p < 0.0001 compared as L/Zi group, $ p < 0.05; $$ p < 0.01; $$$ p < 0.001; $$$$ p < 0.0001 compared as exercise group.

**Figure 3:**
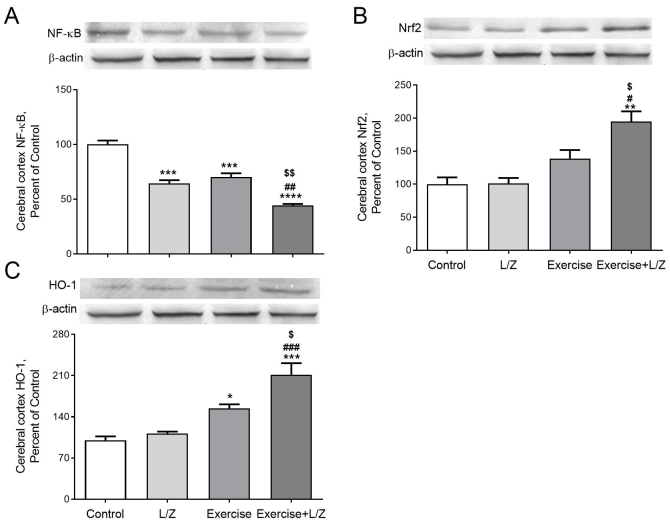
The effects of the lutein/zeaxanthin isomers (L/Zi) with regular exercise on cerebral cortex level of NF-kB, Nrf2, and HO-1 in rats. The level of NF-kB (Panel A), Nrf2 (Panel B), and HO-1(Panel C) is shown in various groups. Data are expressed as a ratio of the normal control value (set to 100%). Each bar represents the mean and standard error of the mean. The intensity of the bands shown in the band was quantified by densitometric analysis and β-Actin was included to ensure equal protein loading. Blots were repeated at least 3 times (n = 3). ANOVA and Tukey’s post-hoc tests were used for comparing the results among different groups. Statistical significance between groups is shown by * p < 0.05; ** p < 0.01; *** p < 0.001; **** p < 0.0001 compared as control group, # p < 0.05; ## p < 0.01; ### P < 0.001; #### p < 0.0001 compared as L/Zi group, $ p < 0.05; $$ p < 0.01; $$$ p < 0.001; $$$$ p < 0.0001 compared as exercise group.

**Figure 4:**
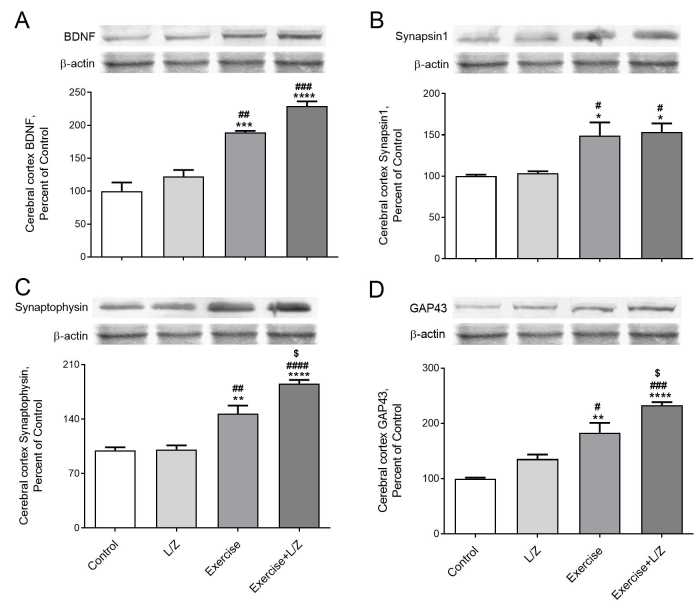
The effects of the lutein/zeaxanthin isomers (L/Zi) with regular exercise on cerebral cortex level of BDNF, Synapsin1, Synaptophysin, and GAP43 in rats. The level of BDNF (Panel A), Synapsin1 (Panel B), and Synaptophysin (Panel C), and GAP43 (Panel D) are shown in various groups. Data are expressed as a ratio of the normal control value (set to 100%). Each bar represents the mean and standard error of the mean. The intensity of the bands shown in the band was quantified by densitometric analysis, and β-Actin was included to ensure equal protein loading. Blots were repeated at least 3 times (n = 3). ANOVA and Tukey’s post-hoc tests were used for comparing the results among different groups. Statistical significance between groups is shown by * p < 0.05; ** p < 0.01; *** p < 0.001; **** p < 0.0001 compared as control group, # p < 0.05; ## p < 0.01; ### p < 0.001; #### p < 0.0001 compared as L/Zi group, $ p < 0.05; $$ p < 0.01; $$$ p < 0.001; $$$$ p < 0.0001 compared as Exercise group.

Rats received L/Zi or performed exercise training showed a significant reduction in muscle NF-κB levels (Figure 2A; p < 0.0001). Compared to control rats, the Nrf2 levels in muscle and (Figure 2B, p < 0.0001) and HO-1 (Figure 2C, P < 0.001) levels were higher in exercised combined with L/Zi rats. A significant reduction NF-κB levels of cerebral cortex were observed in the exercise combined with the L/Zi groups (Figure 3A; p < 0.0001). Compared to control rats, the Nrf2 and (Figure 3B; p < 0.01) and HO-1 (Figure 3C; p < 0.001) levels in the cerebral cortex were higher in exercise combined with L/Zi rats. More importantly, exercise training combined with L/Zi administration resulted in an improving effect on the muscle and cerebral cortex levels of NF-κB, Nrf2 and HO-1 levels. BDNF, synapsin1, synaptophysin, and GAP-43 levels in the cerebral cortex were lower in the control rats than treatment rats (Figures 4A, 4B, 4C, 4D). Compared to the control group, exercise+L/Zi treatment improved BDNF (Figure 4A; p < 0.0001) synapsin1 (Figure 4B; p < 0.05), synaptophysin (Figure 4C; p < 0.0001), and GAP-43 (Figure 4 D; p < 0.0001) levels. 

## 4. Discussion

This work was performed to clarify the effect and possible mechanism of action of L/Zi (a naturally-derived marigold extract) combined with regular exercise on lipid profile, antioxidant properties, and muscle and cerebral cortex transcription and neurotrophic factors, and synaptic protein levels in healthy rats. Serum triglycerides, total cholesterol and lactate concentrations decreased in rats with exercise without supplement or in rats supplemented with L/Zi as compared to control rats. Exercise combined with L/Zi supplementation decreased these parameters with the greatest effect. Many studies reported a decrease in serum concentrations of triglyceride and total cholesterol in exercised rats (2,4). However, no earlier studies examined the effects of exercise combined with L/Zi on serum triglycerides, total cholesterol, and lactate levels in rats to compare with this study. 

L/Zi and exercise treatment reduced the muscle and cerebral cortex MDA levels and increased SOD and GPx activities. L/Zi and exercise treatment decreased the level of NF-κB in muscle and cerebral cortex but increased Nrf2, HO-1, BDNF, and synaptic levels. The most significant increases were found in the combination of exercise and L/Zi treatment. Several studies showed that lutein and zeaxanthin in the tissues, including eye and neural tissues, have many biological effects such as lipid metabolism, antioxidation, and antiinflammation [12,20–22]. These effects may be linked to decreased inflammatory cytokines and a rise in antioxidant enzymes, leading to MDA reduction and subsequent inflammatory responses [23]. Similar to our results, exercise training combined with L/Zi supplementation depressing lipid outline and improving antioxidant status reported in healthy and disease states [12,18,22]. There are numerous studies on using phytochemicals and antioxidants other than L/Zi by exercise [4,18]. Tuzcu et al. [12] reported that L/Zi supplementation decreased insulin and free fatty acid concentration and improved oxidative stress by decreasing MDA levels and increasing the retina’s antioxidant enzyme activities in rats. In another study, healthy term infants orally administered lutein at 12 and 36 h after birth had significantly lower total hydroperoxide levels, an indicator of oxidative stress, and significantly higher biological antioxidant potential in cord blood at 48 h compared to control infants receiving placebo [24]. However, there are also studies showing that lutein or zeaxanthin do not affect oxidative stress. For example, lutein treatment (up to 15 mg/day for 12 weeks) did not have a protective effect against endogenous oxidative DNA damage as indicated by the comet assay or enhance the resistance of LDL-CH to oxidation [25]. 

Transcription factors such as NF-κB and Nrf2 play a critical function in a cellular mechanism involving the prevention/attenuation of oxidative stress originating from biological or environmental stressors [17,26,27]. NF-κB controls the expression of various inflammatory proteins, cell growth, and apoptosis [26]. Nrf2 modulates the expression of more than 200 cytoprotective genes [27]. Recent studies show that Nrf2 shows a vital role in how oxidative stress mediates the useful properties of exercise [27]. In addition, a decrease in NF-κB and an increase in Nrf2 were reported in regular exercise studies [4,18,27]. Though the anti-inflammatory effect of regular exercise [2,28] and L/Zi [12,20,22,23] alone has been well stated, no research has been shown on the outcome L/Zi combined with exercise. Consistent with earlier studies, we found that regular exercise alone improved the inflammatory and antioxidant status by reducing NF-κB expression, an indicator of inflammation and associated diseases, and improving the Nrf2 pathway for antioxidant status [4,18]. Similarly, Tuzcu et al. [12] reported that L/Zi administration decreased the VEGF, iNOS, NF-κB levels and improved Nrf2 and HO-1 proteins in retinal tissues. The present study shows a rise in Nrf2 with a simultaneous increase in HO-1 in L/Zi and exercised rats. Like previous studies, exercise training activates transcription factors, reduces oxidative stress, and increases antioxidant defense [27]. Tan et al. [29] indicated that lutein efficiently downregulated the expression of NF-κB, cyclooxygenase 2, and Nrf2 levels in severe traumatic brain injury rats. Another study reported that lutein or zeaxanthin modulates inflammatory responses in cultured retinal pigment epithelial cells in response to photooxidation [30].

Lutein and zeaxanthin are optimally located in the critical regions for visual [motor and cognitive] processing of the central nervous system. Feeney et al. [31] reported that high plasma lutein and zeaxanthin were independently related to better composite scores in areas of global cognition, memory, and executive function. BDNF is a member of the neurotrophin family mainly active in the hippocampus, cortex, and basic forebrain regions involved in learning, memory, and higher cognitive progressions [32]. It allows the brain cells and synaptic plasticity to grow and survive and is usually reduced after healthy behavior such as physical exercise and psychological stress. It stimulates the growth and survival of brain cells and synaptic plasticity and is usually increased following healthy behaviors such as physical exercise and reduced through times of psychological stress [32]. Synapsins are synaptic vesicle-related proteins that modulate synaptic vesicle exocytosis and play a role in synaptogenesis [33]. GAP-43 protein is a presynaptic membrane phosphoprotein involved in regulating the progress of axons and regulating the formation of new links [34]. SYP is a protein that is complicated in the creation and cycling of the synaptic vesicle, which is highly concentrated in the axonal terminals in neurons, and its level is closely related to the synaptic density [34]. In the present study, L/Zi, and regular exercise increased BDNF, synapsin, GAP-43, and SYP levels in the cerebral cortex. This study showed that L/Zi has a protective characteristic by mimicking synaptic-related proteins and neurotrophic factors to support nerve cell and axonal neurite survival. Since neuroinflammation lowers BDNF and synaptic protein levels, the anti-inflammatory and antioxidant properties of L/Zi can be considered a reasonable mechanism for this effect. Many studies have reported that lutein and zeaxanthin may exert their biological activities such as antiinflammation, neuroprotection, and antioxidation through their influence on reactive oxygen species [12,22]. However, there was no comparative research related to the efficacy of L/Zi on the neurotrophic and synaptic proteins of exercise-trained rats in the previous record, so we cannot evaluate the data of the present study. In an earlier study, it was reported that a positive response to retina and brain supplementation of lutein resulted in proportional increases in systemic BDNF levels [32]. Besides, Lindbergh et al. [35] reported that lutein and zeaxanthin appear to value the neurocognitive role by improving cerebral perfusion, even if consumed for a separate period in late life.

In conclusion, the current study exhibits that a combination of regular exercise and L/Zi enhances exercise capacity, which could be related to inhibition of oxidative stress and modulation of muscle and cerebral cortex NF-κB and Nrf2 pathway. The present study shows that combined L/Zi with regular exercise may reduce neurodegeneration in the cerebral cortex, which may happen by activating neurotrophic factors and synaptic proteins. Moreover, L/Zi can modulate lipid metabolism in healthy rats. 
